# Human-centric infrastructure resilience: Uncovering well-being risk disparity due to infrastructure disruptions in disasters

**DOI:** 10.1371/journal.pone.0234381

**Published:** 2020-06-18

**Authors:** Jennifer S. Dargin, Ali Mostafavi

**Affiliations:** Zachry Department of Environmental and Civil Engineering, Texas A&M University, College Station, Texas, United States of America; US Army Engineer Research and Development Center, UNITED STATES

## Abstract

The objective of this paper is to empirically examine the impacts of infrastructure service disruptions on the well-being of vulnerable populations during disasters. There are limited studies that empirically evaluate the extent to which disruptions in infrastructure system services impact subpopulation groups differently and how these impacts relate to the wellbeing of households. Being able to systematically capture the differential experiences of sub-populations in a community due to infrastructure disruptions is necessary to highlight the differential needs and inequities that households have. In order to address this knowledge gap, this study derives an empirical relationship between sociodemographic factors of households and their subjective well-being impacts due to disruptions in various infrastructure services during and immediately after Hurricane Harvey. Statistical analysis driven by spearman-rank order correlations and fisher-z tests indicated significant disparities in well-being due to service disruptions among vulnerable population groups. The characterization of subjective well-being is used to explain to what extent infrastructure service disruptions influence different subpopulations. The results show that: (1) disruptions in transportation, solid waste, food, and water infrastructure services resulted in more significant well-being impact disparities as compared to electricity and communication services; (2) households identifying as Black and African American experienced well-being impact due to disruptions in food, transportation, and solid waste services; and (3) households were more likely to feel helpless, difficulty doing daily tasks and feeling distance from their community as a result of service disruptions. The findings present novel insights into understanding the role of infrastructure resilience in household well-being and highlights why it is so important to use approaches that consider various factors. Infrastructure resilience models tend to be monolithic. The results provide empirical and quantitative evidence of the inequalities in well-being impacts across various sub-populations. The research approach and findings enable a paradigm shift towards a more human-centric approach to infrastructure resilience.

## Introduction

The impact of natural disasters is often measured by a handful of numbers: the number of fatalities and injuries, the number of homes and buildings destroyed, and the cost of cleanup and repair. However, these measures do not often account for the emotional wounds inflicted on survivors. Furthermore, infrastructure resilience planning needs metrics that are “precise” for measuring both individual system qualities and generalizable in order to inform resource allocations and operations [1,2]. Subjective well-being, defined as ‘a person’s cognitive and affective evaluations of his or her life,’[3] as a human-centric measure of infrastructure resilience allows us to diverge from these standard assessment measures used in evaluating societal impacts of critical infrastructure service disruptions during disasters.

Time and again, natural disasters have tested the resiliency of both built infrastructure and communities. Disruptions in infrastructure services caused by natural disasters have shown to have significant impacts on the well-being of those affected [4–6]. Not only that, but they also cause disproportionate impacts amongst communities and that vulnerability is differential; different people and communities are vulnerable in different ways to different hazards [7]. An increasing number of studies in the disaster literature have focused on the construct of well-being as a central factor in community resilience for [8–15]. However, these studies did not analyze how well-being experience differentiated according to household sociodemographic characteristics as a result of infrastructure disruptions.

Considering the complexity of modern critical infrastructure systems in terms of its interdependencies and external pressures, including increasing demand, aging, and climate change, the risk and severity of disruptions are becoming more likely. Community resilience planning is tasked with ensuring the equitable access and delivery of critical infrastructure system services in cities by reducing the disproportionate risks of service disruptions to the most vulnerable members in a community. However, as our infrastructure systems become "smarter" with the ability to capture more data and make decisions, resilience plans may become less in touch with the individuals and households for whom the resilience strategies exist [7]. Infrastructure and technology-centric approaches generally fail to provide visibility into emergencies caused by infrastructure disruptions in disasters [7]. Likewise, such approaches assume communities of households to be monolithic and are unable to capture the diversity of household characteristics that influence their resilience in the face of disaster [7].

Few empirical studies have focused on the resilience of small groups or units, such as households [16]. The lack of fundamental information about household interactions with infrastructure services in disasters, and more specifically, how these interactions differ with respect to different population attributes. Understanding the disparities in infrastructure disruption impact is key to integrating the needs of diverse populations into planning and prioritization of resilient infrastructure while mitigating impacts to the most vulnerable members of society when infrastructure services are disrupted.

A human-centric approach to infrastructure planning and management is a shift away from conventional engineering approaches which generally focus on the performance and physical failures within systems. From a perspective of resiliency, more focus needs to be directed towards how infrastructure systems make residents feel and whether or not there is an equitable provision of infrastructure services to the community. Having this focus is particularly important in times of disasters where service disruptions can negatively affect human well-being. To address these shortcomings, this paper presents a human-centered approach to empirically analyze the relationships between households and critical infrastructure service disruptions by examining the extent to which disruptions in various infrastructure (e.g., transportation, power, water, and communication) would affect different aspects of well-being (i.e. social and emotional) for different sub-population groups. The results from this analysis aim to emphasize the importance of including different community perspectives and needs in infrastructure resilience planning by providing empirical evidence of impact disparities. Achieving these objectives is critical for advancing the understanding of household network dynamics and integrating human-centric considerations into prioritizing and planning of equitable resilient infrastructure.

### Research scope

A set of human-centric variables (i.e., *measures of social and emotional well-being*) are drawn upon from the Personal Wellbeing Index (PWI) [17] to derive an empirical relationship between sociodemographic factors of households and their subjective experience with disruptions in critical infrastructure services during and immediately after Hurricane Harvey. Overall, the research aims to form the basis of an empirical relationship between human and infrastructure systems while characterizing household-level disparities in impacts due to service disruptions. Households have been selected as the unit of analysis as they are the unit in which network interactions between humans and infrastructure services occur. Accordingly, the characterization of well-being is used to explain the extent to which infrastructure service disruptions influence households differently according to their differential household sociodemographic attributes. This research presents a novel attempt to understanding the role of infrastructure resilience in household well-being outcomes during crisis situations, as well as inequalities in disruption impacts according to different household attributes. More specifically, the analysis is guided by the following research questions:

RQ1: Do infrastructure-disruptions influence well-being impacts in households? If so, what services have the most impacts and on which well-being dimension?

RQ2: Are there disparities among households with vulnerable population groups in terms of well-being impacts caused by infrastructure service disruptions? If so, what sub-populations experience disproportionate well-being impacts for different service disruptions?

To address these questions, a new framework for a human-centric infrastructure service model that conceptualizes the association between humans (in terms of well-being) and infrastructure (in terms of service provisions) is introduced. Secondly, an approach to determining disparities in well-being impacts due to different service disruptions at the household level is discussed and demonstrated using empirical data collected from a household survey and analyzed using correlation analysis. The remainder of this section discusses the knowledge gaps to highlight the point of departure and significance of this study further.

## Literature review

### State of infrastructure resilience approaches

Resilience is “the ability to plan and prepare for, absorb, recover from, and adapt to adverse events” (NRC, 2012) [18]. The goal of infrastructure resilience approaches and analysis is to mitigate negative impacts while ensuring that the “targeted system rebounds to full functionality as quickly and efficiently as possible” [19] (Linkov & Palma-Oliveira, 2017). Societal determinants of risk can be used in resilience models and planning to achieve better economic and social development trajectories [20,21]. Agent-based models have been applied to incorporate complex social measures and household characteristics into infrastructure resilience modeling [22–25]. Methods and tools that do incorporate social considerations for modeling the resilience of infrastructure systems [26,27] either only focus on single dimensions of resilience from a technical standpoint or model the various dimensions separately [27]. They are further limited by narrow outlooks on the service population, neglecting various household-level attributes that might influence the service quality experience across different households. In order for resilience planning and risk reduction planning processes in infrastructure systems to consider societal dimensions of service disruptions, it is essential to understand the human well-being impacts. Disaster impact on humans has been studied from economic, psychological, and physical health perspectives. However, such studies do not entirely focus on infrastructure systems or their services and do not capture the impact of disaster-inflicted service outages on households sheltering in place. Hence, empirical research such as the work presented in this paper is necessary for determining appropriate community resilience metrics that account for the interaction between society and built infrastructure [26].

### Gaps in research

The role of infrastructure systems and the services they provide in community resilience and the importance of understanding and reducing disruption impacts have been established by interdisciplinary fields [28–30]. Measuring the impact of infrastructure services on human well-being and the extent of risk and uncertainty involved in the operations of infrastructure systems is imperative for creating both resilient infrastructure and communities [31,32]. More particularly, the need to know how to integrate the needs of diverse populations in planning and prioritization of resilient infrastructure. This knowledge will help to improve social inequities, and as a result, foster more resilient communities. It then becomes clear that there is a need for human-centric approaches to infrastructure resilience planning and modeling. Until now, socioeconomic measures that are typically used in studies rely on GDP, mortality rates, and patient data, which are not sufficient in capturing the differential well-being of shelter-in-place households before and during disasters. Disaster research has been able to at a high level, identify that minority groups are more prone to the impacts of disasters. However, the research does not explicitly relate these outcomes to the infrastructure systems that enable them nor specify the influence of infrastructure disruptions on the well-being of these vulnerable groups. Very few, if any, infrastructure resilience or disaster recovery models exist that represent variables of community well-being or public health [33]. This limitation is mainly due to the lack of empirical information that specifies the relationship between infrastructure disruptions and various elements of human well-being for different sub-populations. A couple of studies [34,35] have used network analysis and social media data to examine changes in transportation and wireless infrastructure systems in the context of disasters. Song et al. (2018) [36] developed a resilience model to measure the resilience level of different areas during typhoons based on social, economic, infrastructural, and natural components. This study did not, however, look at a direct relationship between infrastructure disruptions and household impacts. Román et al. (2019) [37] used spatial analysis to track outages and recovery times of power infrastructure during Hurricane Maria and linked these measures to census-based demographic characteristics of residents. Similarly, this study focuses on a single infrastructure system.

### Well-being as a measure for minimizing social inequality in disaster risks

Disasters have long-term and serious effects on the emotional and social wellbeing of the populations impacted [38]. Additionally, higher levels of well-being have been shown to be an indicator of greater resilience to disaster impacts and the ability to recover. In order to increase levels of societal and individual wellbeing, there needs to be a reduction in socioeconomic inequalities [39–41]. If the underlying purpose of human-centered approaches to infrastructure resilience is to improve social equity in communities, then appropriate measures need to be adapted. Measures of societal progress need to be able to understand the diverse experiences and living conditions of people [42]. Existing protocol, standards, and guidelines for engineering performance governing infrastructure design rarely include the socioeconomic impacts of infrastructure service disruption. Often, measures of social impacts of disaster typically attempt to quantify the impact in terms of economic loss [7], mortality, or medical cases [42]. For example, Burrus et al. [43] and Santos et al. [44] understand workforce recovery and how it couples with critical infrastructure availability. Such measures, however, do not provide sufficient assessment of the living conditions that people in communities experience.

In the context of infrastructure resilience and disaster, measuring subjective well-being in and of itself can draw more attention to the needs of different sub-populations within a city and towards potential action-oriented solutions [45]. In fact, integrating measures of well-being in infrastructure resilience assessments can shift the focus from "systems" to "people." Several countries and cities have already started working in this space, measuring subjective well-being households in official statistics that are intended to drive policy decisions [46] and to assess and inform public policy and other action plans [42,47–49]. Nevertheless, the current resilience planning and risk reduction processes in infrastructure systems have yet to adopt measures of well-being to inform investment, resource allocation, and prioritization decisions and policies.

### Vulnerable populations and resilience

During and after disasters, "vulnerable populations require more assistance and are the least capable of taking care of themselves and generally live in the oldest and most hazardous buildings" [30]. Variations in socio-demographic attributes, access to resources, expectations, and norms cause specific households to endure more significant impacts as a result of infrastructure disruptions during and after a storm. Isolating vulnerable persons during and following a disaster event actually creates an increased *dependency burden* on infrastructure services when it is least afforded [50]. Numerous studies have clarified the heightened vulnerability of population groups such as the elderly, children, linguistic minorities [51]. and low-income households during and in the aftermath of a disaster event. Research over the last several years [52–53] has analyzed the social impacts of transportation disruptions following severe weather events based on measures of accessibility [46]. While the studies were distributed across different geographic areas and urban settings, all **c**oncluded that impacts on the communities differed demographically [7]. Studies have shown that racial and ethnic minorities are less likely to evacuate in times of disasters [7, 52,54] and more likely to live in areas predisposed to environmental hazards and risks [55]. Studies have shown that particular age group populations are also disproportionately exposed to weather-related hazards [56–58]. Silverman and La Greca (2002) [58] studied the impact that children experience during and after a disaster. Based on their findings, the community outcome of a disaster generally show that minority youth have a greater chance of reporting high levels of PTSD symptoms; this then leads to their conclusion of minority youth having a much more difficult time recovering than those of non-minority youth. During Hurricane Sandy, researchers found that power outages had different mental health impacts of counties and individuals of lower socioeconomic status [59]. These studies suggest the importance of an evaluation of disparities in well-being impacts for vulnerable subpopulations exposed to infrastructure service disruptions.

### Conceptual framework

**Fig 1:**
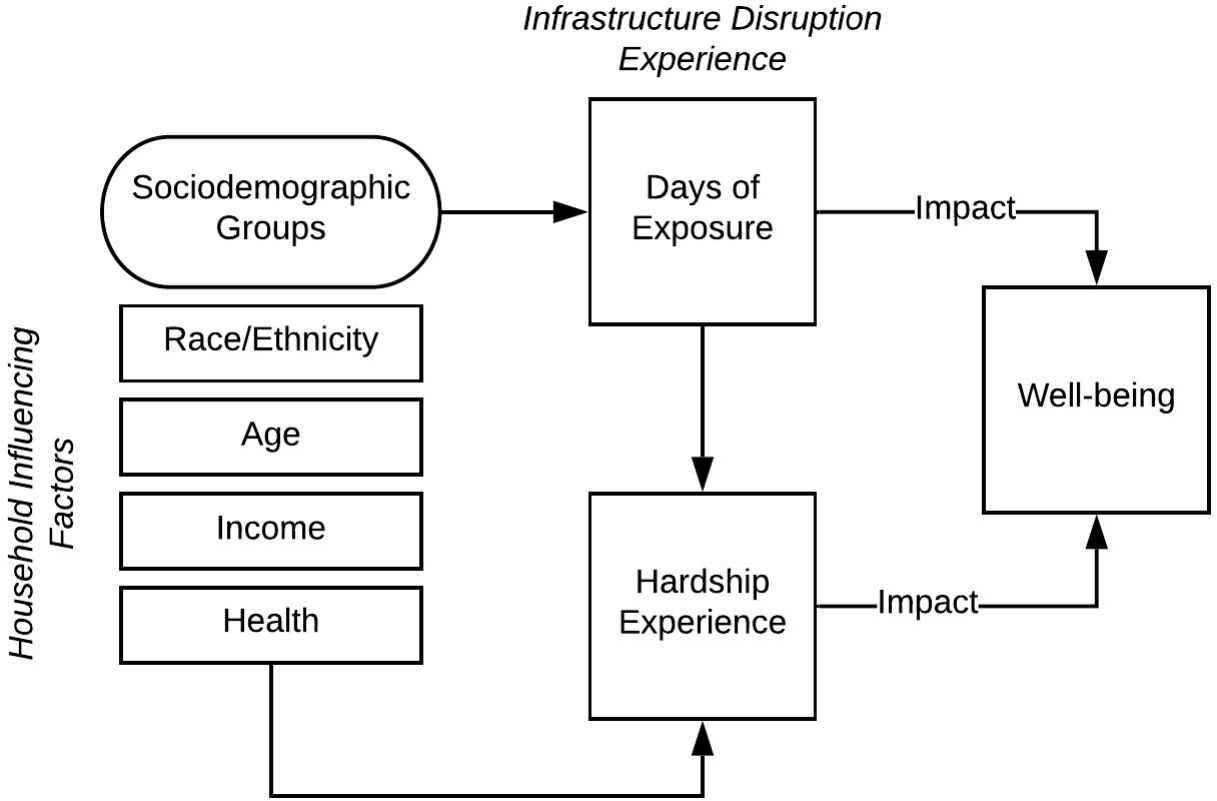
Infrastructure resilience and well-being framework.

This paper proposes an infrastructure resilience and well-being disparity framework (Fig 1) to conceptualize the association between humans (in terms of well-being) and infrastructure (in terms of service provisions). The connection between well-being and infrastructure disruptions is supported by the capability approach framework [60]. According to Sen’s capability approach, the provision of resources enables people to develop capabilities that help them achieve ‘functioning’s,' or in other words, an enhanced state of well-being [61]. We use this idea to bridge the measures of hardship experience due to infrastructure disruption and well-being. In this context, the infrastructure system aims to provide households with services that enable them to develop or do specific tasks (jobs, school), with the underlying goal to maintain or improve well-being. This approach is suitable for expressing non-tangible damage caused by natural hazards and disasters, such as the subjective experiences of individuals and households.

Fig 1 presents the conceptual model of infrastructure resilience and household well-being disparity. The framework summarizes and relates two components (households and infrastructure) using three constructs (well-being, sociodemographic characteristics, and infrastructure services). In this framework, household well-being (in relation to infrastructure) is determined based on two elements: (1) the extent of service disruptions (days of exposure to service outages, and (2) the extent of hardship experience due to service disruptions. The extent of hardship is used as an indicator for examining the nature of experience related to a service disruption. A household’s hardship experience is influenced by various factors such the socio-economic characteristics, preparedness, and access to resources. In this study, only socio-demographic characteristics are considered since the goal of the analysis is to examine the presence and extent of disparities in well-being impacts among various sub-populations. This section here on out defines the variables and pathways depicted in Fig 1.

### Well-being as a human-centric component

According to Doorn et al. (2018) [26], societal well-being depends on the interconnection and feedback between physical infrastructure and the ability of individuals to adapt to disruptions. Disasters have long-term and serious effects on the emotional and social wellbeing of the populations impacted [31]. Additionally, elevated states of well-being have been associated with greater resilience to disaster impacts and the ability of communities to recover. States of emotional or mental well-being, which are key determinants of one’s ability to cope with the normal stresses of life [61–62], and in particular, the stresses induced by disasters. Therefore, it is the social and emotional aspects of well-being that are emphasized and analyzed with the presented framework. This framework defines the onset of a disaster event and the resulting infrastructure disruptions as extrinsic factors that have the potential to influence a household’s level of well-being. Self-reported subjective well-being impact measures are drawn upon from existing well-being and mental health, a component of well-being, assessments to quantify the social and emotional well-being of households within one month of Hurricane Harvey. These measures are presented and discussed in the proceeding section (Table 1).

**Table 1:** Adapted measures for well-being impact assessment.

Well-being Measure	Survey Question
*Helplessness*	How often did you find yourself or a household member helpless (one month after Hurricane Harvey)?
*Anxiousness*	How often did you or a household member feel anxious, worried or nervous (one month after Hurricane Harvey)?
*Upsetting thoughts*	How often did you or a household member have upsetting thoughts and feelings related to the storm or the damage it caused (one month after Hurricane Harvey)?
*Safety*	How much did your household‚ experience with Hurricane Harvey make you or members of your household feel less safe and protected in your daily life (one month after Hurricane Harvey)?
*Depression*	How much did your household experience with Hurricane Harvey make you or members of your household feel depressed or restless (one month after Hurricane Harvey)?
*Daily life tasks*	How much did your household‚ experience with Hurricane Harvey lower your ability to do your daily life tasks such as working or dealing with others (one month after Hurricane Harvey)?
*Feeling distant*	How much did your household‚ experience with Hurricane Harvey make you or members of your household feel distant or cut off from other people (one month after Hurricane Harvey)

Given the interdependencies of modern lifestyles and infrastructure services as addressed in the previous section, it is then apparent how lack of service or diminished quality in service can impact the well-being states of people. The impact of a natural disaster is often measured by a handful of numbers: the number of fatalities and injuries, the number of homes and buildings destroyed, the cost of cleanup and repair. It does not often account for the emotional wounds inflicted on survivors. Well-being as a human-centric measure of infrastructure resilience allows us to diverge from these standard assessment measures used in evaluating societal impacts. While the self-reported well-being measures examined in this study can be indicators of actual mental illness (clinical depression), the purpose is not to identify whether or not there are more incident cases of mental illness.

#### Infrastructure service hardship experience

Critical infrastructure includes systems and assets, which in the event of incapacity or destruction, would have a debilitating impact on the functioning of society from a perspective of public health, national security, and economic security [30]. While there are 16 infrastructure systems deemed critical by the federal government, research tends to focus on just five of them: energy (particularly electric power), water, wastewater, transportation, and telecommunications systems. Food and solid waste services are not typically included in research involving critical infrastructure systems even though the significant and long-lasting damages disasters have caused to them in the past and potential public health hazards they can create [63–66]. Disadvantaged communities often suffer disproportionately from the impact of waste facilities [66] and racial minorities are more likely to live in closer proximity to waste facilities [66].

Access to food retailers becomes limited due to disruptions in other supporting critical infrastructure systems, including electricity, potable water, and transportation [67]. Similarly, vulnerable populations such as children, the elderly, ethnic minorities, and low-income households are disproportionately affected by food security [68,69]. During Hurricane Katrina, access to supermarkets declined for all census tract neighborhoods but was primarily limited for African American tracts, which 71 percent less likely to have access to a new supermarket [70]. In the case of Hurricane Harvey, disruptions to foodservice systems (broken refrigeration units and supply chains) led to the expansion of urban food deserts. To remedy the disruption in food availability and access, the Supplemental Nutrition Assistance Program (SNAP), was expanded to nearly 600,000 households affected by the storm [71].

In socioeconomic research, economic hardship has been shown to thwart well-being in households and individuals [72–74]. Based on this assumption supported by theoretical research, it is proposed that hardship experience due to infrastructure disruptions also impacts household well-being. Hence, in this study, we use hardship as a proxy variable for determining the relationship between well-being and infrastructure service disruptions. Disparities among sociodemographic groups are often studied in public health and epidemiological research in the context of health equity and are defined as occurring when a population group has a disproportionate share of health burden [75]. The *infrastructure resilience and well-being disparity model* applies the same concept and definition of disparity: when one subgroup population has disproportionate experience in well-being impact as a result of the exposure and experience with infrastructure service disruptions. The higher impact of infrastructure disruption experience indicates a more significant negative well-being impact.

#### Days of exposure

In Esmalian et al. (2019) [22], the authors found no significant disparity in days of exposure to hardship experience across various subgroups for electricity services in Hurricane Harvey. This finding indicates that exposure to service disruptions was not significant for different socio-demographic groups. For that reason, we do not link days of exposure to sociodemographic factors. Furthermore, the study found a positive correlation between days of exposure to hardship experience. Hardship experience is used as a proxy variable of infrastructure disruption experience to draw a connection to well-being experience. The household influencing factors only focus on sociodemographic groups so that we can analyze whether or not sociodemographic play a role in well-being experience. Sociodemographic characteristics are hypothesized to influence the extent of hardship experienced, which is also determined by the days of exposure. On the other hand, as the households experienced more service losses (more days of a power outage), they experience more hardship as shown by the positive correlation between the interruption and self- reported hardship. However, we hypothesize the sensitivity of hardship experience (and the subsequent well-being impacts) to the duration of service disruption varies for different sub-populations and various infrastructure services.

#### Household factors influencing disparity

Social vulnerability in the context of disaster management emerged from the realization that socioeconomic factors affect community resilience [74]. In disaster events, infrastructure disruptions frequently cause or exacerbate many types of socioeconomic impacts, including health, social, economic, and environmental consequences [7]. Vulnerable populations referred to in this framework have been derived from the Social Vulnerability Index (SVI) [75] and include households who are racial or ethnic minorities, children, elderly, socioeconomically disadvantaged, underinsured or those with certain medical conditions. SVI was developed to assist in disaster planning and public health practitioners in identifying high-risk communities during hazards or recovering from disasters [75]. It uses U.S. Census Bureau data to determine the social vulnerability at tract-level based on 15 social factors grouped by four related themes: Socioeconomic status, Household Composition/Disability, Minority Status/Language, Housing/Transportation [75]. While sociodemographic factors influence the extent of hardship, and ultimately the well-being experienced, it is recognized that life events (i.e., disasters and changes to critical infrastructure services) have the potential to be detrimental to health and well-being [76].

## Materials and methods

This research was approved by Texas A&M University IRB: IRB2018-0459M. No consent was obtained because the data were analyzed anonymously. The study is centered around the critical infrastructure outages affecting Harris County residents during Hurricane Harvey. Hurricane Harvey was a Category 4 storm that made landfall in Texas on August 25th, 2017. Harvey led to severe rainfall and mass flooding throughout the state. The proposed well-being framework is used to identify areas of risk disparity due to infrastructure service disruptions within a population. The survey design, data measures, and analytical approach are described. Moreover, we utilize empirical data from Hurricane Harvey to test the proposed framework in answering the proposed research questions in the context of critical infrastructure system disruptions due to disaster.

### Survey design

**Fig 2:**
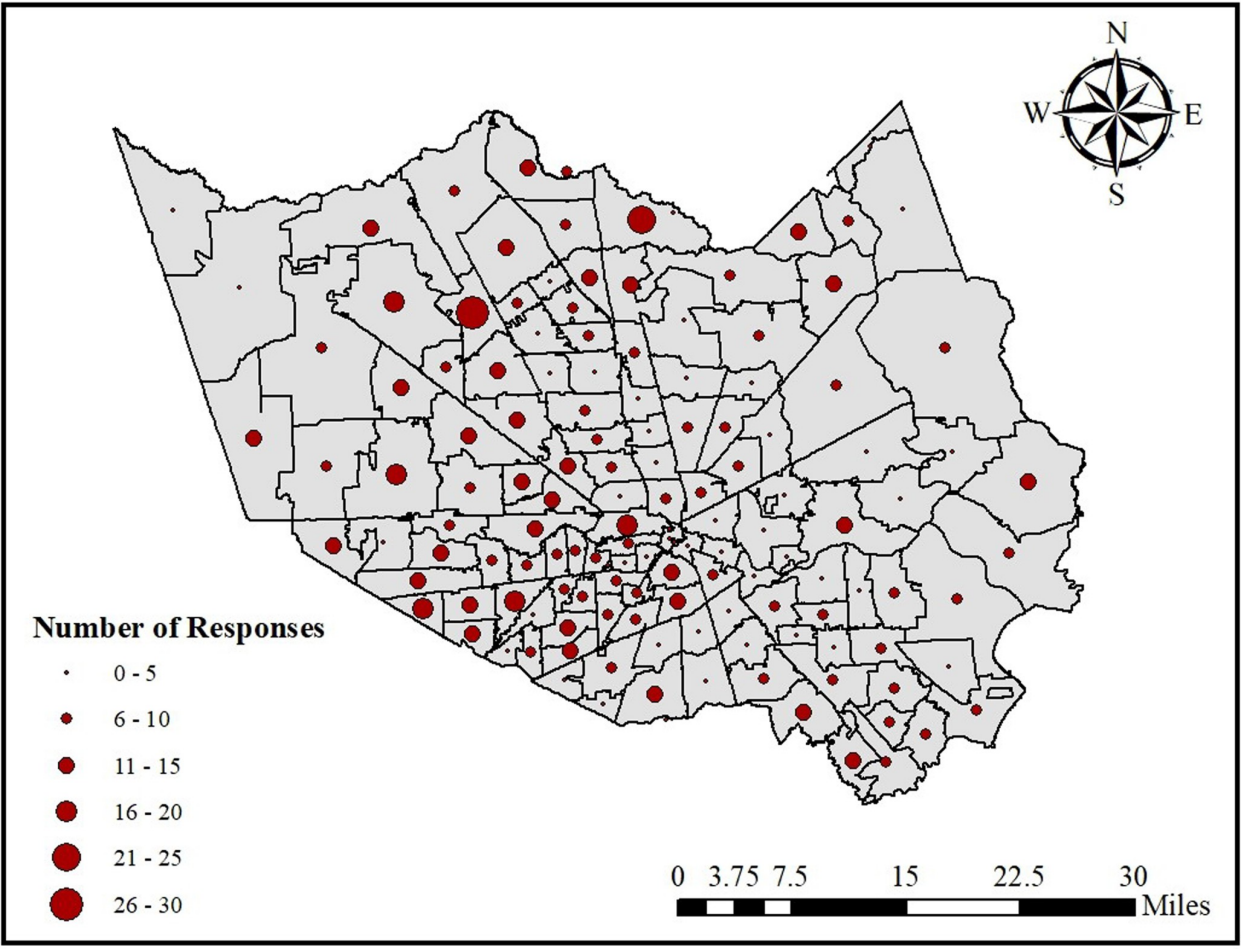
The distribution of households in the study area; Harris County, Texas.

Hurricane Harvey made landfall on Texas in late August 2017, impacting all 4.7 million inhabitants of Harris County, the most populous county in Houston and in Texas. Record-breaking rainfall wreaked havoc on Houston’s infrastructure systems and households making it one of the costliest disasters in U.S. History, after Hurricane Katrina. All 22 of Houston metro’s major freeways were flooded and impassable during the storm while nearly 300,000 households lost power [77]. Empirical data were collected from households in Harris Country, Texas to gather information on household exposures to infrastructure gaps, hardship experiences due to infrastructure service disruptions, and changes to well-being states as a result of the disaster experience according to race/ethnicity, age, socioeconomic status, pre-existing health conditions (disability, chronic health). Questions measuring well-being impact focused on experienced or “hedonic” well-being [78]. A web-based survey was deployed between April and May 2018 through Qualtrics, a survey company that matches respondent panels with demographic quotas. In order to represent the vulnerable population groups in the study area, the authors provided quotas created from U.S. Census Bureau data to draw a sample from Harris County based on age, race/ethnicity, income, and health status. All participants in the survey were required to be of age 18 years or older. An initial sample of 47 questionnaires was first distributed to check the quality of the questions, and a review of the results determined that the survey was ready for the complete data collection. The purpose of the data was to highlight the trends in vulnerable population group experiences with infrastructure disruptions during a disaster event. As suggested by Lindell (2008) [79] the degree to which sample means and proportions are representative of the study area population is less important than having enough demographic diversity to provide an adequate test of the relationships in the presented correlation analysis. A total of 1081 household samples were collected from 140 of the 145 zip codes in Harris County (Fig 2) According to power analysis, this is a sufficient number of responses to conduct inferential statistics that systematically examine associations within the survey data. Those with incomplete responses and those that had evacuated their households before Hurricane Harvey landed were eliminated from the analysis, narrowing the analyzed sample to 837 households. The focus of this research is on households sheltering in place during a disaster; this discretion, therefore, excludes households that evacuated prior to Harvey’s landfall. Harris County was selected particularly because mandatory evacuation orders were not issued to its residents. Within Harris County, only one city issued a voluntary evacuation order [80]. Several coastal counties along the Gulf Coast were ordered to evacuate [80] which would have made these counties inadequate for our study. The rationale for this selection was that, for the people who evacuated and had to move to shelters or other places, the relevance of infrastructure service disruptions becomes of secondary importance since they have already lost their shelter (the primary place in which infrastructure services are utilized).

### Data and measures

This section presents the measures derived from the household dataset that are used as empirical inputs of the proposed infrastructure-well-being framework.

#### Well-being

This study uses self-reported subjective emotional and social well-being impact measures modified from the Personal Wellbeing Index (PWI) [17] and from a literature review on prominent surveys administered post-disaster that focused on the aspects of social and emotional well-being evaluation of impacted people [12,14–15, 81–84]. The PWI focuses on measuring an individual’s satisfaction according to a specified set of seven core domains: standard of living, personal health, achieving in life, personal relationships, personal safety, community connectedness, and future security. The domains, *standard of living* and *future security* are not specifically addressed in this study because they do not directly contribute to social and emotional dimensions of well-being that this study primarily focuses on. Additionally, while personal health is one of the domains of the PWI, it does not specifically measure states of emotional or mental well-being, which are key determinants of one’s ability to cope with the normal stresses of life [12], and in particular, the stresses induced by disasters. The authors drew upon the most cited emotional determinants of well-being found in post-disaster mental health assessments: *feeling depressed*, *having upsetting thoughts*, and *feeling anxious*. Furthermore, the inclusion of multiples determinants of emotional well-being will allow the authors to draw connections and determine the aspects of well-being most relevant to disaster experiences and infrastructure disruptions.

Subjective indicators of household well-being are derived from seven survey questions used to measure the social levels of households within one month after the experience of the hurricane event (Table 1). They are self-reported, measured in a five-point Likert-scale ranging from None at all (= 1) to A great deal (= 5). According to the OECD [41] and standard practice in the psychology field [81,82], using multiple items in a scale is preferred so that a broad construct, such as an adverse effect, is measured categorically. The improved reliability of subjective Likert well-being scales as compared with single-item measures, can thus potentially be attributed to their ability to reduce the impact of random error [42]. Table 1 outlines the well-being components included in the final assessment and the survey questions used to collect the measurements.

#### Hardship

Self-reported hardship due to disruptions in infrastructure services was measured in a five-point Likert-scale ranging from None at all (= 1) to A great deal (= 5) for the following question: Households were asked: “What was the extent of overall hardship experienced due to X outages/interruptions posed by Hurricane Harvey?” Self-reported hardship is used as a proxy for examining the experience of households due to service disruptions. The extent of hardship experienced is correlated with both the exposure to disruptions, as well as the socio-demographic characteristics of households. Greater exposure to hardship experiences would lead to more significant well-being impacts. Hence, the combined effects of the extent of hardship experience and the duration of exposure are used to examine well-being impacts on households.

#### Household sociodemographic factors

Household sociodemographic characteristics may describe vulnerable groups that are often at a disadvantage while preparing for, responding to, and recovering from disaster events [7]. For this analysis and to maintain consistency with the elements of social vulnerability index (SVI), households have been classified into subgroups according to reported age groups in the household, ethnic identity, health status, and income level. The survey did not differentiate between white Hispanic and non-white Hispanics. Additionally, the ‘*other’* racial category represents households that identified as mixed-race or ethnicity in addition to Pacific Islanders and Native Americans. Pacific Islanders and Native American households were grouped into the *Other* category because of the low population samples. Most statistical analyses require sample sizes to be greater than or equal to 10. The income group levels were divided into three brackets (low, middle, high), according to recent census data on median household income in Texas [85]. Sub-categories were combined, as shown in Table 2.

**Table 2:** Measurement of the influencing sociodemographic factors of household well-being disparities.

Sociodemographic Domains		Survey Measures & Encoding
**Age**	*Under 10 years*	*Household has child under 10 (Yes (= 1) or No (= 0)*
	*between 11–17*	*Household has child between 11–17 (Yes (= 1) or No (= 0)*
	*Over 65*	*Household has elderly resident (Yes (= 1) or No (= 0)*
	*Reference group*	*Households without children or elderly residents (Yes (= 1) or No (= 0)*
**Income**	*Low Income*	*$25K - $50K; (= 1)*
	*Middle Income*	*$55K-$99K; = (2)*
	*High Income*	*$100K+; (= 3)*
	*Reference group*	*High Income*
**Ethnic Identity**	*Black*	*(Black = 1*, *Non-black = 0)*,
	*Hispanic/Latino*	*(Hispanic/Latino = 1*, *Non-Hispanics/Latino = 0)*,
	*Asian*	*(Asian = 1*, *Non-Asian = 0)*,
	*Other*	*(Other = 1*, *Non-Other = 0)*,
	*Reference group*	*(White = 1*, *Non-White = 0)*,
**Health**	*Chronic Disease*	*Household has resident with condition*: *Yes (= 1) or No (= 0)*
	*Disability*	*Household has resident with condition*: *Yes (= 1) or No (= 0)*
	*Reference group*	*No health condition reported*: *Yes (= 1) or No (= 0)*

Identifying reference points is significant for determining disparities across subgroup populations, as their nature cannot be understood unless the point relative to which they are measured is identified [86]. A reference point is defined as "the specific value of a rate, percentage, proportion, mean, or other quantitative measures from which a disparity is measured" [86]. For *race or ethnicity* subgroups, the reference point has been defined as the group that represents the most substantial proportion of the population [87]. Disparities can also be measured relative to a standard or target [86]. For example, the reference group for income level groups has been determined by the most ideal or favorable income level group (above $100,000). As for health status and age, reference points were determined by households without any reported vulnerable age groups (elderly or children) or health condition (disability or chronic disease). Table 2 summarizes how each sociodemographic factor is measured in the statistical analysis and specifies the reference group for each sociodemographic domain. The set of groups are mutually exclusive and attributed to binary values, apart from income groups which have been encoded as numeric values of 1 through 3, representing low, middle, and high income.

### Analysis

The relationship between households and infrastructure disruptions is analyzed from three dimensions: 1) the household sociodemographic characteristics, 2) Reported Infrastructure hardship experience, and 3) household well-being impact. Statistical analysis using R programming was performed on the filtered dataset to determine well-being risk disparity, with the level of significance set at p < 0.05. According to the first dimension, the data was divided for each sociodemographic group (Table 2), resulting in 15 datasets. Spearman rank-order correlation analyses were performed between each well-being dimension and each critical infrastructure service disruption experience for each dataset representing the 15 possible sociodemographic subgroups. The Spearman correlation analyses have been used as a non-parametric alternative to linear regression and Pearson correlations when dealing with two measurement variables where one or both variables may generally not be distributed [88].

**Fig 3:**
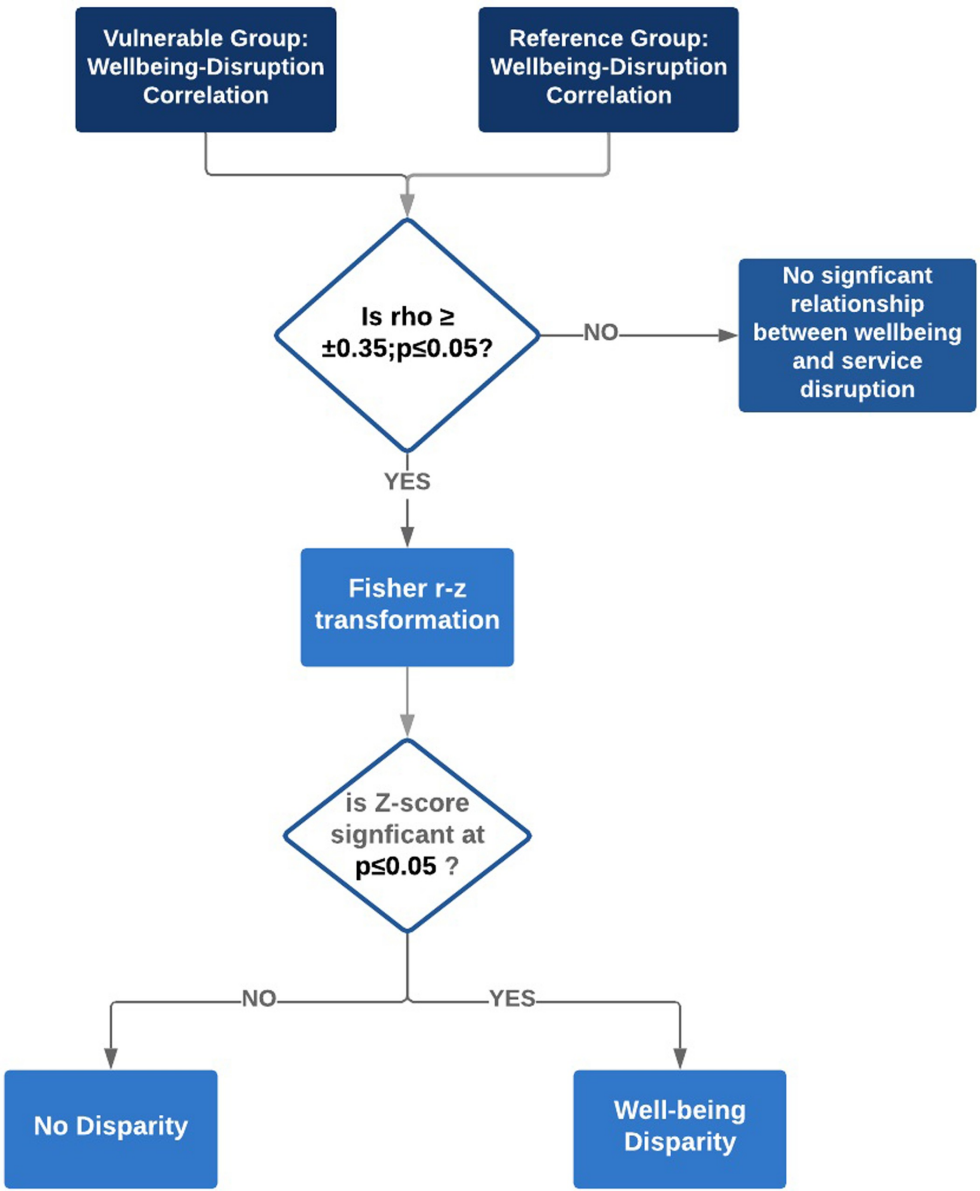
An analytical approach for measuring subgroup well-being disparity.

Disparities among the identified household influencing factors, as presented in Table 2, were determined by comparing the *rho* values of the subgroups and their respective reference groups using Fisher z-tests (Fig 3). These tests measured whether or not the difference in subgroups (vulnerable populations) and reference groups (non-vulnerable population) were statistically significant. Given that the p-value is less than 0.05, results from the Fisher z-tests would signify the presence of well-being impact disparity associated with the household’s sociodemographic characteristic(s).

Tests for significance were conducted using the R *cocor package* [89]. Akoglu, H. (2018) [90] recommends that a specific coefficient should be interpreted as a measure of the strength of the relationship in the context of the posed scientific question, as opposed to clear rules for interpreting coefficient values. Based on the recommendations of literature [86], coefficient values below 0.35 were classified as weak. Correlation coefficients above .35 were considered moderate to high correlation. Trends are identified to characterize services by well-being dimension, and also to identify which subgroup was associated most with which well-being component due to associations with which infrastructure service.

A disparity is determined when there is a significant difference between the coefficient of the vulnerable group and the reference population, and the coefficient is greater than or equal to 0.35, indicating a moderate (either positive or negative) association between well-being impact and infrastructure service disruption. In the context of risk disparities, it is essential to select appropriate metrics to characterize resilience and selection of acceptable risk levels or thresholds. Doorn et al., 2018 [26] suggests that using average values as risk thresholds can be misleading as they can mask important trends and variations within a population sample. Average values were therefore avoided as they may reflect a case in which all individuals had roughly the same impact, or a case in which some individuals were not impacted but a portion of the population was severely impacted [91].

## Results

The sociodemographic composition of the households represented in the dataset are summarized in Table 3. Since the focus of this research is at the household level, specific information about the individual responders were not collected apart age and county of residence to determine their eligibility for participating in the survey.

**Table 3:** Sociodemographic characteristics of households in the survey.

Household Sociodemographic Identification		Frequency in Survey	% of All Households
**Ethnic Identity**	White	491	59%
	Hispanic or Latino	95	28%
	Black or African American	160	15%
	Asian	38	4%
	Other	52	6%
**Income**	< $25,000	188	22%
	$25,000–$49,999	184	22%
	$50,000–$74,999	185	22%
	$75,000–$99,999	110	13%
	$100,000–$124,999	81	10%
	$125,000–$149,999	61	7%
	> $150,000	27	3%
**Health**	Disability	146	17%
	Chronic	238	28%
**Age**	Under two years	37	4%
	Between 2–10	105	13%
	Between 11–17	120	14%
	Between 18–64	575	69%
	65+	257	31%

Total households = 837.

This study analyzed the relationship between humans and infrastructure from three-dimensions: 1) a household’s sociodemographic characteristics, 2) reported hardship due to infrastructure disruptions, 3) reported household well-being. Statistically significant disparities in well-being experience across all household subgroups were found concerning all infrastructure service disruptions apart from Electricity (this is probably because of the non-extensive power outage in Harris County during Harvey). The Spearman correlation coefficients associating well-being dimensions with infrastructure disruption experience were statistically significant for groups across all racial, age, health, and income subpopulations (p<0.01) apart from Asian households. This finding alone implies that the association between well-being and service disruptions is not due to an experimental-wise error.

The claim that certain population groups experience higher risks in disasters and service disruptions is empirically-backed by the results of this study’s correlation analysis: the identified vulnerable population groups tend to have stronger correlations to well-being impact due to infrastructure disruptions compared to their reference group. However, not all of the differences were proven to be statistically significant.

Table 4 presents sociodemographic groups that experienced statistically significant disparity in well-being impact due to infrastructure disruption. It includes the dimension of well-being and the infrastructure service in which disparity was prevalent, the Spearman correlation coefficient of the association for the sociodemographic group and reference group, as well as the z-score resulting from the Fisher z-score coefficient difference tests. All coefficient values represented in this table are significant at p > 0.01.

**Table 4:** Subgroup populations experiencing disparity by well-being dimension and infrastructure service.

Subgroup	Well-being Dimension	Infrastructure Service	Rho	Reference Group Rho	Fisher z-score
Black/African American	Upset	Transportation	0.502***	0.323***	2.28
	Helplessness	Solid Waste	0.451***	0.293***	1.93*
	Depression	Food	0.513***	0.224***	3.56*
	Anxiety	Food	0.425***	0.248***	2.12*
	Upset	Food	0.420***	0.212***	2.45*
	Safety	Food	0.470***	0.232***	2.86*
	Helplessness	Food	0.502***	0.236***	3.27*
	Daily tasks	Solid Waste	0.420***	0.241***	2.07*
	Daily tasks	Food	0.500***	0.224***	3.38*
	Distance	Food	0.460***	0.285***	2.09*
Asian	Anxiety	Food	- 0.352*	0.285***	3.23*
	Safety	Water	- 0.243	0.319***	3.18*
Other	Safety	Solid Waste	0.645***	0.351***	2.57*
	Anxiety	Solid waste	0.597***	0.319***	2.31*
	Daily Tasks	Solid Waste	0.612***	0.241***	3.00*
	Upset	Solid	0.528***	0.312***	1.70*
	Depression	Solid	0.579***	0.308***	2.20*
	Distance	Solid	0.573***	0.338***	1.93*
	Helplessness	Solid	0.563***	0.293***	2.16*
Low-income	Distance	Transportation	0.398***	0.243***	2.00*
	Daily tasks	Solid Waste	0.328***	0.158**	2.09*
	Distance	Solid Waste	0.369***	0.198***	2.16*
Middle Income	Daily tasks	Solid Waste	0.348***	0.158**	2.35*
	Distance	Solid Waste	0.454***	0.198***	3.32**
Children (11–17 years)	Safety	Water	0.445***	0.281**	1.87*
No Health	Distance	Communications	0.344	-	2.11*
	Daily Tasks	Communications	0.349	-	2.07*
Chronic Health	Depression	Solid Waste	0.425***	0.270**	2.30*
Disability	Distance	Water	0.489***	0.330***	1.97*
	Distance	Food	0.462	0.285***	2.12*

Based on the rho values alone, well-being dimensions measured by the *"Ability to do daily life tasks*" and "*Feeling distant or cut off*" appear to be more strongly associated with well-being impact as compared to the other dimensions considered in this study. *“Feeling depressed”* or *“feeling upset”* did not have as significant or influential associations with infrastructure disruptions. The association between well-being and infrastructure disruptions was disproportionately stronger for Black and African American households compared to both the reference group (White) and other racial groups. On the other hand, infrastructure service disruptions did not have a significant impact on the well-being of Asian households, for which most rho coefficients were below 0.20 and or negative. For example, a negative correlation between disruptions in Food services and “*feeling anxious”* was found (rho = -0.352, p<0.01). The association between “*difficulty doing daily tasks”* and disruptions in electricity services is the only significant positive rho coefficient found in Asian households (0.336, p<0.01).

In the remainder of this section, findings specific to each subgroup population, followed by infrastructure service are presented in detail.

### Well-being impact disparities among subpopulations

#### Race and ethnicity

Table 5 highlights the strongest associated well-being dimensions and service disruptions contributing found for each ethnic group. In general, for all racial and ethnic groups, changes in well-being appear to be most associated with most disruptions in food access, followed by transportation, and solid waste services. Only households identifying as *Black* and *Other* were associated with disproportionately greater well-being impact due to service interruptions, with respect to the reference population. For Black households, the results indicate moderately high correlations between all well-being components and disruptions in Transportation services followed by Food (0.40<rho<*0*.*51*, p = 0.001). Although weaker with respect to other infrastructure services, Communication service disruptions appear to have had disproportionately impacted the well-being of African American households (rho =, reference rho =). Households identifying as *Other*, interestingly, showed significant correlations between disruptions in Solid Waste services and all well-being dimensions, most notably Safety (rho = 0.644).

**Table 5:** Characterization of racial and ethnic groups by the strongest associated well-being and infrastructure disruption.

Race/Ethnicity	Well-being Dimension	Infrastructure Service
*Black*	Depression, Daily-tasks	Food, Transportation
*White*	Anxiety	Transportation
*Asian*	Daily-tasks	Electricity
*Latino*	Daily-tasks	Communications
*Other*	Safety	Solid Waste

Conversely, correlation analysis conducted for Asian households suggests minimal well-being impact due to disruptions in infrastructure, indicated by both negative correlations and insignificant coefficients at p<0.05. It is interesting to note, however, the association between Electricity and Daily tasks (0.33627), was the only non-negative coefficient higher than 0.30 at p<0.05. The correlation between disruption and well-being impact for the reference group population was moderately low, where coefficients of all well-being-disruption relationships were below 0.40, p = 0.001. The strongest association for White households was found between Anxiety and disruptions in Transportation services (0.3844195, p = 0.001). Latino households, similar to White households, had moderately low correlations. However, while the difference was not found to be statistically significant from the reference group, the association between communication disruptions and “*difficulty with daily tasks”* as well as solid waste disruptions and *“feeling distant”* are notable for Latin households.

#### Income

Table 6 shows the strongest associated well-being dimensions and service disruptions contributing to the well-being impact for each income group. In general, the correlation between well-being impact and infrastructure disruptions for low-income households was stronger compared to middle and high-income groups. High-income households are characterized by low to mild correlations where disruptions in transportation and association with the dimensions, “*feeling upset”* (0.3971156, p<0.001) and *“feeling anxious”* (0.389103, p<0.001) were the highest rho values. As for low-income households, the associations between all well-being dimensions and transportation disruptions were comparably stronger. All well-being dimensions for low-income households fell between 0.399 and 0.43 p<0.001, where the dimensions “*difficulty with daily tasks”* (0.4314670, p<0.001), and *“feeling anxious”* (0.4373609, p<0.001) were the highest rho values. For Middle-income households, solid waste disruptions appeared to have the most significant impact on well-being ("*feeling distant*," 0.4539269, p<0.001).

**Table 6:** Characterization of Income groups by the strongest associated well-being and infrastructure service.

Income Level	Well-being Dimension	Infrastructure Service
*Low*	Daily-tasks, Anxiety	Transportation
*Middle*	Distance	Solid Waste
*High*	Upset	Transportation

#### Age

Table 7 shows the strongest associated well-being dimensions and service disruptions contributing to the well-being impact for each Age group. Households without any children or elderly residents were more likely to experience well-being impacts due to disruptions in transportation and solid waste, whereas “*feeling distant*” was the strongest associated well-being. Weak correlations between well-being and infrastructure disruptions were observed within the Elderly subgroup population. Households reporting at least one or more Elderly resident have a stronger positive relationship between “*feeling helplessness”* and all infrastructure disruption categories (rho < 0.30, p<0.001). However, no coefficient exceeds 0.40 concerning all disruption and well-being categories. Based on the results, it is apparent that infrastructure disruptions alone did not contribute to significant impacts on the well-being of households with elderly residents. However, higher rho values tend to be associated with transportation disruptions. *"Safety"* and "*difficulty with daily tasks”* characterized the infrastructure disruption experience of households with children between the age of 11 and 17.

**Table 7:** Characterization of Age groups by the strongest associated well-being and infrastructure service.

Age Group	Well-being Dimension	Infrastructure Service
*Under 10*	Distance, Safety	Solid Waste
*11–17*	Safety; Daily-tasks	Water, Communications
*18–64*	Distance	Solid Waste
*Over 65*	Helplessness	Transportation

Interestingly, it does not appear that one infrastructure service dominates over another, in terms of its ability to impact household well-being. The strongest associations occur between disruptions in water infrastructure and the well-being dimension, *safety”*, in addition to disruptions in communications and *“difficulty with daily tasks*.*”* Households with children under ten years follow a similar trend in well-being impact; however solid waste disruptions appear to have a wider-spread impact: all measures with well-being have coefficients greater than 0.35 (p<0.001). The most significant relationships among households with children under ten were found between solid waste disruptions and “*feeling distant”* (0.4653714, p<0.001), followed by solid waste disruptions and “*safety”* (0.4558314, (p<0.001).

#### Health

Table 8 shows the strongest associated well-being dimensions and service disruptions contributing to the well-being impact for each Health group. For households with disabled residents, well-being is correlated most with feeling distant; moderate coefficients were determined for water, food, solid waste, and transportation disruptions (in order from strongest to weakest coefficients). Furthermore, disruptions in solid waste services appeared to have a broader impact on reported well-being: *feeling distant*, *unsafe*, *anxiety*, *and difficulty doing daily tasks* were all moderately correlated with solid waste service disruptions. Households without reported health conditions experienced stronger impacts with respect to certain well-being dimensions than those with Chronic illness or Disability, as shown in Table 5.

**Table 8:** Characterization of Health groups by the strongest associated well-being and infrastructure service.

Health Group	Well-being Dimension	Infrastructure Service
*Chronic Illness*	Distance, Safety	Solid Waste
*Disability*	Distance	Water, Food
*No pre-existing health condition*	Upset, Anxiety, Daily Tasks	Transportation, Electricity

Households with disabled occupants had more frequent and moderate associations with feeling distant with respect to disruptions in water (0.4892208, p<0.001) and food infrastructure (0.4617829, p<0.001). The broad well-being impacts of solid waste disruptions are also found from the correlation analysis for disabled households, indicated by the frequency of rho values higher than 0.35. Households with chronic health conditions followed similar trends to those of disability, but values were milder. Overall, coefficients for households reporting disability were stronger compared to households with no reported health conditions or chronic-health households.

### Influence of different infrastructure disruptions on well-being dimensions

#### Transportation

**Fig 4:**
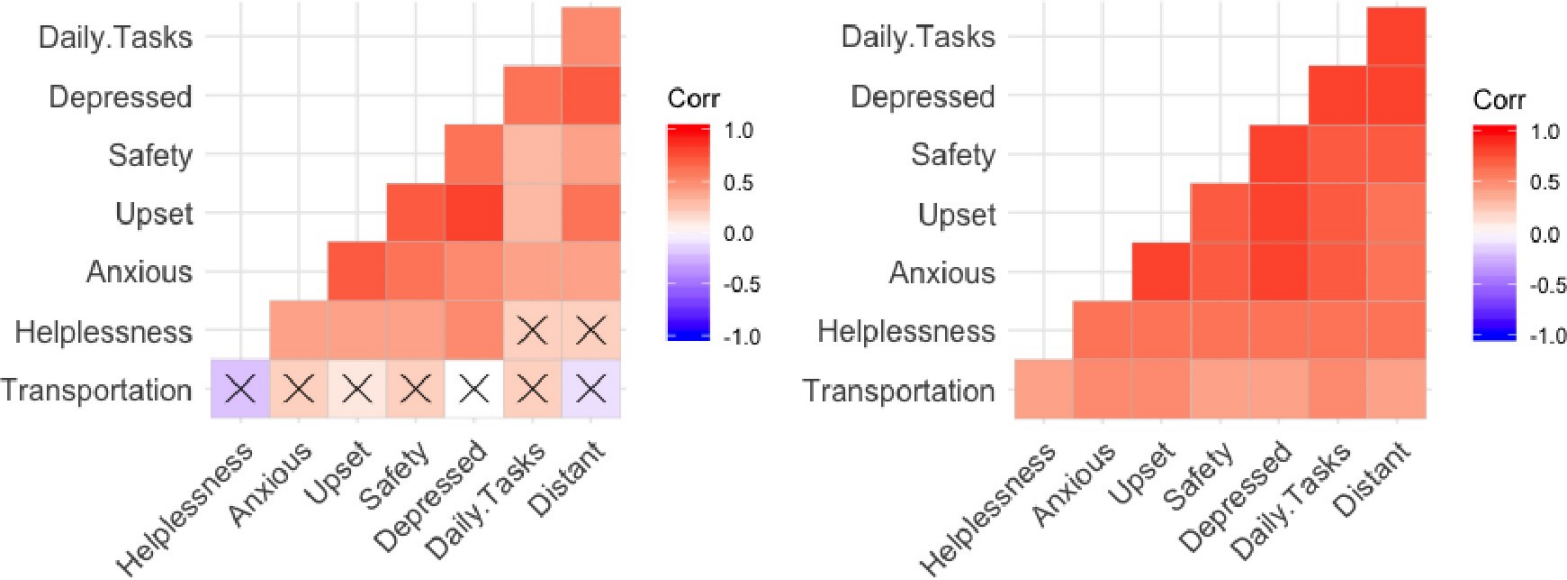
Well-being and transportation disruption experience correlation matrix for Asian Households (Left), Black households (Right). X's signify p>0.05.

Of the 15 subgroups (including the reference groups for each domain), 14 groups apart from households identifying as Asian, the rho coefficients measuring the relationship between *feeling anxious* and disruption in transportation were greater than or equal to .35. Six subgroups contained a rho value of .40 or higher (Low income, Black, Hispanic or Latino, Under ten years old, Chronic Health, No Health Condition). Despite the common theme of *anxiousness* with respect to transportation disruptions, the strongest associations occurred within the analysis of Black households, where the strongest correlation occurred with the dimensions *feeling upset* (0.50233, p<0.001), followed by *feeling anxious* (0.4549, p<0.001). Both are statistically different from the reference group, signifying a significant disparity in the well-being impact due to disruptions in transportation, influenced by racial and ethnic minority status. Other dimensions of well-being that were impacted by disruptions in transportation services include: *feeling distant*, *difficulty carrying out daily tasks*, and *feeling helpless*. The least impacted households by transportation services were households identifying as Asian (Fig 4), Disabled, over 65, and *Other* race.

#### Solid waste

Similar to transportation services, solid waste disruptions appear to have a negative impact on the well-being of all subgroup populations, in which *feeling distant*, *safety*, and *daily tasks* resulted in the strongest rho. The highest rho value was the result of the correlation with *safety* (0.65 p<0.001) followed by *daily tasks* (0.61, p<0.001), with respect to households identifying as “Other” race or ethnicity. The difference from the reference group is statistically significant, indicating a racial disparity in well-being risks due to disruptions in solid waste services.

#### Electricity

Less than half of the subgroup categories were associated with the same well-being dimension, so we are unable to categorize electricity disruptions with a single or particular cluster of well-being dimensions. The strongest rho value is the measure between electricity disruption experience and *daily tasks* for Black or African American households (0.4335 p<0.001). Interesting to note are the negative correlations reported by Other and Asian groups.

#### Food

We observe a similar trend for Food services: less than half of the subgroup categories were associated with the same well-being dimension, so it is not possible to categorize electricity disruptions with a single or particular cluster of well-being dimensions. The disruptions in food services were most strongly correlated to ‘feeling depressed' in *Black or African American* households (rho = 0.51287, p<0.001), followed by helplessness and daily tasks (0.5018 and 0.5003, both at p<0.001).

#### Water

Interruptions in water services is associated more strongly by ‘feeling helpless’, for eight subgroups (Low income, High income, Black, Hispanic or Latino, 11–17 years, between 18–64, No health condition, Disability), followed by ‘difficulty doing daily tasks’ (Low income, middle income, Black, under 10 years, 11–17, No health condition, Disability). The strongest relationship, however, is between households with disabled members and ‘feeling distant’ (rho = 0.48, p <0.001).

#### Communications

Less than half of the subgroup categories were associated with the same well-being dimension; therefore, we are unable to characterize communication disruptions with a single or particular cluster of well-being dimensions. We see a moderate correlation between the difficulty of doing daily tasks, in which five groups (Low income, Black, Latino or Hispanic, 11–17 years old, and no existing health condition group), coefficients are greater than or equal to 0.35, p<0.05. Only a small number of the coefficients were above 0.35, signifying, in this case, that communication disruptions did not result in significant impacts on household well-being during and following the disaster. Also, this may indicate that there was not an extensive disruption in communication services among the studied households. In the case of Hurricane Harvey, we can infer that communication infrastructure, in comparison to other infrastructure systems, was resilient against hurricane-related damages. Despite this, the disparity in experiences was still found: Black households (0.46, p<0.001) and Latino households (0.4, p<0.001) were more strongly associated with communication disruptions compared to both the reference group and other ethnic groups, similarly, age group 11–17 (0.43 p<0.001).

## Discussion

This effort is among the first studies to systemically and empirically evaluate the social inequalities related to well-being risks in the context of infrastructure resilience. The results of this study provide empirical grounding and evidence of the inequitable state of risks due to infrastructure disruptions. In the context of this study area and Harris County in Harvey, disruptions in *transportation*, *solid waste*, *food*, *and water* infrastructure services resulted in more significant well-being impact disparities as compared to *electricity and communication services*. It is likely that the planning and preparation efforts taken by communication service companies led to limited disruption and rapid restoration that buffered the impact of disruptions on households during the storm. In fact, only 5% of the wireless networks in Harris County experienced outages [92], where 283,000 households lost wired phone services at the peak of the outage in contrast to more than 3 million phone lines in Hurricane Katrina and one quarter of wireless networks in Superstorm Sandy [92]. The utilities prepared by topping off all generators ahead of the storm and purchasing spare fuel and having refueling trucks on standby at specific locations [93]. In case of damages to fiber lines, microwave technology was used to temporarily bridge gaps where fiber lines were disconnected from cell towers to communication centers. Power outages during Harvey never exceeded 350,000 customers at any time, compared to millions that lost power in Hurricane Ike and Hurricane Katrina [94].

Furthermore, the average reported days of electricity disruptions in the sample households is 0.790 days, while the average duration of wireless and internet service outages were 1.18 and 0.845 days, respectively. This is in contrast to the average five days of household reported duration in transportation service interruptions. This can be an example of how strategic resilience planning, as well as retrofit and mitigation investments for disaster, can improve the resiliency of infrastructure systems and minimize the impact on households during a disaster, contributing to the household and community resiliency.

Based on the analysis, factors such as their ethnicity and household income affect the amount of tolerability that a household can emotionally and mentally withhold when experience hardships due to service disruptions. Using the correlation analysis, this study discovered disparities in well-being experience due to disruptions in infrastructure services, primarily with respect to racial or ethnic groups. In particular, the results showed the strongest correlations between their well-being and infrastructure disruptions, namely Food and Transportation, for households identifying as Black or African American. Households identifying as *Other* ethnicities, which comprised of Native American, Pacific Islander, and Multiracial families experienced significant disparity in well-being experience concerning Solid Waste services. While not statistically significant according to the fisher z-testing analysis, the results for other minority groups and vulnerable group populations had overall stronger associations between well-being impact and disruptions compared to the reference group (non-vulnerable) population. These findings suggest that the risks of infrastructure service disruptions disproportionately affect the well-being of vulnerable populations. The remaining of the discussion is organized concerning the research questions guiding the analysis.

### The association between disruptions and well-being dimensions

**Fig 5:**
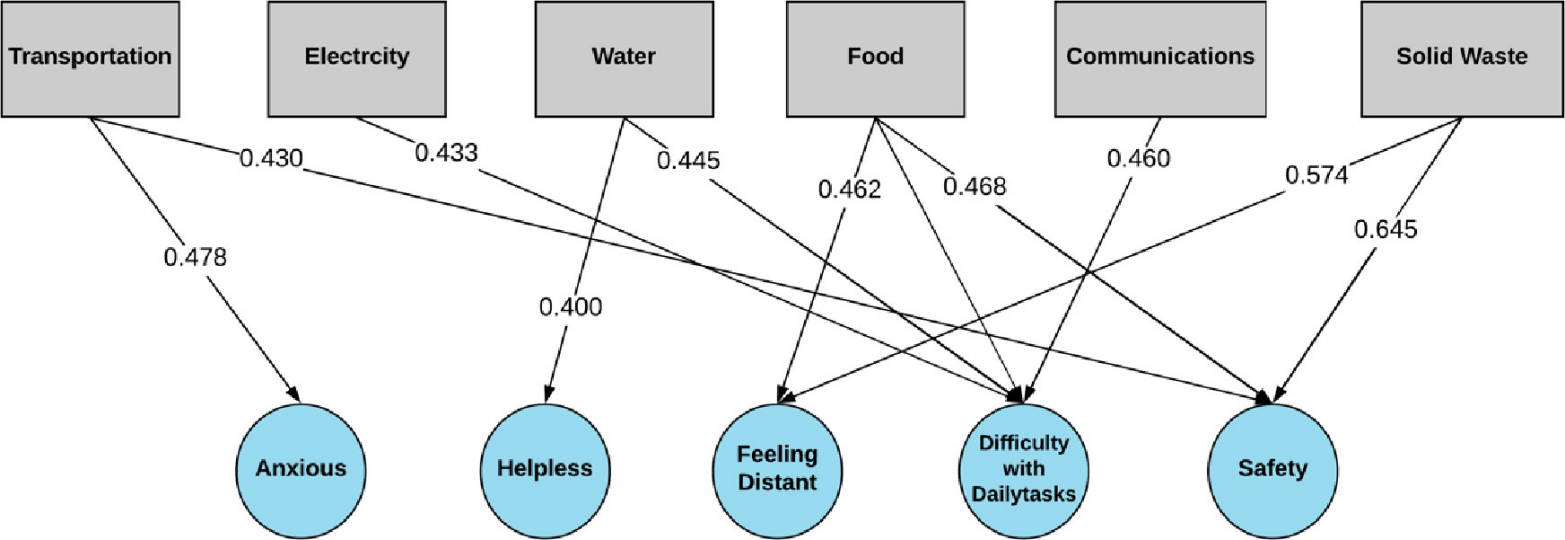
Service disruption characterization. The characterization of service disruptions by average Spearman’s rho and most frequently associated well-being dimension according to all subgroup populations is shown. Service disruptions are frequently associated with difficulty with daily tasks, followed by Safety and Feeling Distant. Well-being dimensions “Depressed” and feeling “Upset” did not appear to be associated frequently with infrastructure disruptions.

Fig 5 is a visual representation of the well-being characterizations of the six critical infrastructure systems examined in this study. Different sub-group populations experience different levels of well-being risks due to disruption in infrastructure services. For example, the correlation analysis shows that households identifying as Black and African American experienced greater well-being impact compared to other sub-group populations. Also, well-being impacts found in households identifying as Black and African American had stronger associations with disruptions in food, transportation, and solid waste services. Secondly, different components of well-being were found to be associated more strongly with certain infrastructure services. In general, service disruptions were more likely to results in households feeling helpless, having difficulty doing daily tasks, and feeling distance from their community. In some subpopulation groups, feeling more distance or disconnected from their community was significantly correlated with disruptions in solid waste services. A systems analysis would be needed to investigate the influencing factors of such relationships.

The moderate levels of correlation coefficients hint towards additional factors that influence well-being changes as a result of infrastructure disruptions during disasters. Those factors influencing well-being risk disparities include variations in the level of preparedness, perceived risk of disruptions, and ability to adjust to disruptions. The influence of these factors is not considered in the current analysis and will be the next step for this research.

The results from the correlation analysis confirm that infrastructure disruption impact on communities is indeed heterogeneous. In the context of Hurricane Harvey, vulnerable groups have a higher sensitivity to service disruptions. Using Fisher z-score tests, significant disparities in well-being experience were identified across racial or ethnicity, income level, health status, and age group (Table 5). However, the race and ethnicity of households appear to hold more considerable influence on disparities in well-being, compared to other factors surveyed in this study, such as income, age, and health.

The insignificance in the correlation between well-being impact and infrastructure hardship experience for Asian households and the more significant correlations for Black households is very telling and indicative of the inequalities that exist in infrastructure service disruption impacts. The minimal disparities and difference in well-being impact across the age group categories confirm what many researchers in behavioral science and psychology have already found, but specific to the context of infrastructure disruptions. Outside the context of infrastructure disruptions, some studies theorize that the relationship between age and well-being is a relatively stable one, with a tendency to increase slightly with age [95]. Others argue that well-being is not actually influenced by chronological age [96] and that other factors such as physical health status and conditions of living are more significant in determining the well-being of people [97]. Similarly, Khumalo et al. [98] found that older adults are more likely to have higher mental well-being than those younger than them. While the findings from these studies are not specific to disasters or infrastructure system disruptions, they help to shed light on the outcomes of this analysis. In the case of this study, households with elderly residents were less susceptible to well-being impact due to Electricity and Communication Services, and for the other infrastructure services, elderly households often fared better compared to their reference group. For households with elderly occupants, disruptions in infrastructure services did not appear to have strong correlations to changes in well-being.

More significant variation and disparity in well-being impact among households grouped by income level were anticipated. However, the results rejected the presence of well-being impact disparities for households with different income levels. Mirroring the findings of Stewart-Brown et al. (2015) [99] on subjective well-being and income in a general context, well-being associations were relatively constant among all income groups apart from the categories displayed in Table 5. Their study found no evidence of a dose-response relationship for income and high mental well-being. In general, the chance of low mental well-being was increased along with reduced income, but those in the second lowest income bracket were more likely to have low mental well-being than those in the highest (although those in the lowest income bracket showed no difference to the highest). While those in the highest income bracket were more likely to have high mental well-being than those in lower brackets, the four lowest brackets showed very similar levels of high mental well-being. This finding also points out the role of other influencing factors such as preparedness, previous experiences, and risk perception.

### Well-being disparities among subpopulation groups

Certain infrastructure services cater more towards specific demographics, mainly communication services. The well-being of Households with older children (11–17) or no children was correlated more strongly to disruptions in communication services, in comparison to the Elderly and households with children under the age of 10 years. Research has shown that older adults are slower in adopting new technologies than younger adults [100]. Older adults (60–91 years) were less likely than younger adults to use computers and the internet [101]. Likewise, younger children under the age of 10 years are less likely to use communication services directly. Young adults and adults may have a higher dependency on communication services due to education needs and work-related purposes. Changes to communication service can thus harm their well-being state, more so than other age groups as the correlation analysis suggests.

Just as particular infrastructure services have more significance to different population groups, certain well-being dimensions are more associated with different infrastructure services. This information provides more insight into which infrastructure systems were more resilient in terms of population impact. In the context of Hurricane Harvey, Electricity, Communications, and Food services were weakly correlated to well-being impact for most population groups, apart from African American households. This might be an indication that these systems were not severely disrupted. There is also a tendency of well-being impacts due to infrastructure service disruptions to be more related to social needs of households, as opposed to emotional needs. This distinction reinforces the idea that infrastructure systems support the social fabric and connectivity of communities.

Resilient infrastructures and resilient communities go hand in hand. Some well-being dimensions are more impacted than others due to different infrastructure service disruption’s impact. This is an important observation because it indicates the ways through which infrastructure service disruptions impact humans. *‘Feeling distant or cut-off’* and ‘*feeling difficulty in doing daily tasks’* means that disruptions in services interrupt the household's ability to feel like productive members of their community. Being distant from the community and unable to make contributions lead to communities being more disconnected, and as a result, less resilient in the face of calamities and disruptions. While the relationships in this study are associative rather than causative, the findings show disparities in well-being impacts of infrastructure disruptions for different vulnerable sub-populations.

### Future work

The primary focus of this study is to empirically examine the relationship between infrastructure disruptions and households’ well-being, which is an understudied area in the field of infrastructure resilience. It is out of the scope of our paper to assess why certain infrastructure systems cause more impact in certain well-being dimensions than others. Future studies can examine the underlying mechanisms that influence the well-being impact disparities identified in this study due to various infrastructure service disruptions.

While the study was able to show an empirical relationship between well-being impact and certain sociodemographic characteristics of households and that there is a disparity that is related to sociodemographic characteristics, the study does not look into the specific factors that influence this apparent disparity. Further analysis would focus on narrowing in on the latent influencing factors that predispose vulnerable populations to greater well-being impact compared to other households. The future studies of the authors will investigate the association of other social factors such as gender, as well as characteristics particular to disaster situations such as preparedness, previous experience, expectations, and social capital on the well-being impacts of infrastructure disruptions.

Furthermore, this study does not analyze the combined effects of infrastructure disruptions on household wellbeing. For example, disruptions in transportation services and road access may have influenced African American households’ access to food services at more significant levels compared to other services and non-African American households. Similarly, certain significant associations between particular well-being dimensions and infrastructure service disruptions, such as ‘feeling distant’ and solid waste service disruptions, need to be investigated further to uncover why certain disruptions impact particular well-being dimension more than others. Looking at the combined effects of infrastructure systems from a systems-of-systems perspective would help community planners pinpoint fundamental infrastructure interactions that contribute to higher impact in vulnerability in certain population groups.

### Concluding remarks

This paper addressed critical gaps in empirical knowledge surrounding household-infrastructure disruption interactions. The purpose of this paper was to advance the understanding of household-infrastructure system dynamics and break new ground in our understanding of social inequality of the risk impacts due to service disruption. The empirical analysis presented in this study confirms that there are household level influencing factors that are associated with the differential impact of service disruptions on households during a disaster event. Disparities in well-being impact were most prevalent among racial minority groups related to disruptions in transportation, solid waste, and food services.

Furthermore, the inclusion of well-being as a measure of the household level impact allowed us to determine which aspects of well-being infrastructure disruptions are more strongly associated. Infrastructure disruptions associated with the greatest disparity also tended to have strong correlations to multiple well-being dimensions. Similarly, subgroups experiencing impact disparity, such as Black and Other households, tend to have high associations with multiple well-being dimensions.

To the best of authors’ knowledge, no prior studies have been attempted to systematically assess the impact of multiple infrastructure disruptions and well-being dimensions. As a result, multiple novel perspectives and understanding of household-infrastructure disruption dynamics were uncovered through this study to enable an equitable resilience approach in infrastructure systems. The results from this research have interdisciplinary applications and implications by encouraging the integration of social dimensions into disaster planning and prioritization of infrastructure systems. As a result, the inequity in impacts that sub-groups experience when different infrastructure services are disrupted can be examined more effectively, and the extent to which infrastructure service disruptions create disproportionate risks for different sub-populations can be understood.
